# Transcriptomic Analysis of the Combined Effects of Methyl Jasmonate and Wounding on Flavonoid and Anthraquinone Biosynthesis in *Senna tora*

**DOI:** 10.3390/plants13202944

**Published:** 2024-10-21

**Authors:** Saemin Chang, Woo-Haeng Lee, Hyo Ju Lee, Tae-Jin Oh, Si-Myung Lee, Jeong Hwan Lee, Sang-Ho Kang

**Affiliations:** 1 Department of Agricultural Biotechnology, National Institute of Agricultural Sciences, RDA, Jeonju 54874, Republic of Korea; saeminchang@gmail.com (S.C.); ju950114@naver.com (H.J.L.); tataby@korea.kr (S.-M.L.); 2 Division of Life Sciences, Jeonbuk National University, 567 Baekje-daero, Deokjin-gu, Jeonju 54896, Jeollabuk-do, Republic of Korea; jhwanlee90@jbnu.ac.kr; 3 Department of Life Science and Biochemical Engineering, Sun Moon University, Asan 31460, Republic of Korea; kain832@naver.com (W.-H.L.); tjoh3782@sunmoon.ac.kr (T.-J.O.)

**Keywords:** *Senna tora*, transcriptomics, flavonoid, anthraquinone, chalcone synthase, wounding, methyl jasmonate

## Abstract

Jasmonates, including jasmonic acid (JA) and its derivatives such as methyl jasmonate (MeJA) or jasmonly isoleucine (JA-Ile), regulate plant responses to various biotic and abiotic stresses. In this study, we applied exogenous MeJA onto *Senna tora* leaves subjected to wounding and conducted a transcriptome deep sequencing analysis at 1 (T1), 3 (T3), 6 (T6), and 24 (T24) h after MeJA induction, along with the pretreatment control at 0 h (T0). Out of 18,883 mapped genes, we identified 10,048 differentially expressed genes (DEGs) between the T0 time point and at least one of the four treatment times. We detected the most DEGs at T3, followed by T6, T1, and T24. We observed the upregulation of genes related to JA biosynthesis upon exogenous MeJA application. Similarly, transcript levels of genes related to flavonoid biosynthesis increased after MeJA application and tended to reach their maximum at T6. In agreement, the flavonols kaempferol and quercetin reached their highest accumulation at T24, whereas the levels of the anthraquinones aloe-emodin, emodin, and citreorosein remained constant until T24. This study highlights an increase in flavonoid biosynthesis following both MeJA application and mechanical wounding, whereas no significant influence is observed on anthraquinone biosynthesis. These results provide insights into the distinct regulatory pathways of flavonoid and anthraquinone biosynthesis in response to MeJA and mechanical wounding.

## 1. Introduction

Plants are constantly faced with biotic and abiotic stresses and developed defense systems to respond to stresses [1]. Secondary metabolites are one of the means by which plants survive environmental stresses [2,3]. Plant hormones regulate the biosynthesis of numerous secondary metabolites [4,5,6]. Plant hormones are divided into several sub-classes according to their chemical structures and comprise auxins, gibberellins, ethylene, cytokinins, abscisic acid, jasmonic acid (JA), salicylic acid, brassinosteroids, and strigolactones [7].

Jasmonates are phytohormones whose accumulation responds to many biotic and abiotic stresses. They play an essential role in plant development, stress responses, and secondary metabolite regulation as essential signaling molecules. Jasmonates include all intermediates and products of the JA biosynthesis pathway and its metabolites. In addition to JA, the most studied jasmonates are methyl jasmonate (MeJA) and jasmonyl-isoleucine (JA-Ile), which are formed by jasmonic acid *O*-methyl transferase (JMT) and by JASMONATE RESISTANT 1 (JAR1), respectively, in the cytoplasm. MeJA, a methyl ester of JA, is volatile and can easily diffuse into the air and mediates plant-to-plant communication in response to biotic and abiotic stresses without having to rely on transmission through the vasculature [8,9]. JA-Ile is a biologically active isoleucine conjugate that regulates many stress responses and developmental responses. JA and MeJA are particularly important in defense responses to necrotrophic pathogens and wounding by insects, as well as diverse stages of plant development [10,11,12,13,14,15]. Collectively, jasmonates, whether produced within plants or applied exogenously, regulate the expression of many genes involved in plants development and responses to various stresses.

*Senna tora* (L.) Roxb. is an annual shrub belonging to the family Fabaceae (subfamily Caesalpinioideae). It is also known as *Cassia tora* and generally distributed throughout India, Pakistan, Sri Lanka, and China [16,17,18] and is used as an herbal medicine in these and nearby countries. This plant offers medicinal benefits on account of its anti-diabetes [19], anti-dermatitis [20,21], anti-constipation [22], and anti-bronchitis properties. These various effects are due to numerous active secondary metabolites that are anti-inflammatory, antibacterial, or antioxidant [17,23,24,25]. Such diverse medicinal ingredients are distributed in various parts of this plant, including the leaves, roots, and seeds [26,27,28]. Among various metabolites, flavonoids tend to be found in relatively larger amounts in leaves and anthraquinones in seeds [28,29]. Both the flavonoids and anthraquinones are polyphenolic compounds, and their medicinal effects are mostly attributed to their antioxidant property [30,31]. They modulate reactive oxygen species (ROS) levels as radical scavengers and protect plants from harmful UV irradiation as a UV protectant [32]. In particular, kaempferol and quercetin, two flavonols, attracted much research attention because they are medicinally useful [33,34,35].

Previously, we showed that mechanical wounding accelerated the kaempferol and quercetin accumulation in *S. tora* leaves [36]. In this study, we comprehensively analyzed the global expression patterns of genes involved in metabolism and the specific secondary metabolite production (flavonoid and anthraquinones) under the combined effects of MeJA and wounding. This study provides valuable insights into the regulatory pathways of flavonoid and anthraquinone biosynthesis in response to both biotic and abiotic stresses.

## 2. Results and Discussion

### 2.1. Identification of DEGs in Response to MeJA Treatment and Wounding

The sequencing libraries derived from total RNA extracted from *S. tora* leaves before wounding (T0) and after 1 (T1), 3 (T3), 6 (T6), or 24 (T24) h of MeJA application produced 33,502,278–46,846,336 trimmed reads—after processing raw reads. The clean reads encompassed between 3,358,299,940 and 4,690,843,228 bases with a percentage of reads with a minimum quality score of Q30 of 96.1% to 97.1%. We mapped the clean reads to the *S. tora* reference genome [37], returning expression information for 18,883 genes (mapping ratio of 95.5–98.0%, Supplementary Table S1) from the three replicates for each time point. Thus, we obtained expression data for about 41.7% of all protein-coding genes annotated in the genome (out of 45,268 genes).

**Figure 1:**
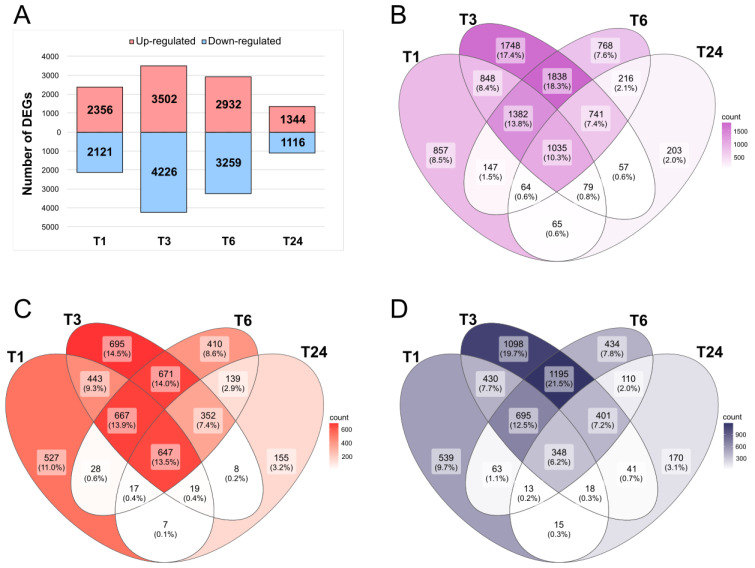
Differential gene expression analysis among time points after methyl jasmonate and wounding treatments in *S. tora*. (**A**) Number of upregulated and downregulated DEGs at each time point relative to T0 (before wounding and spraying) samples. Venn diagrams showing the extent of overlap among total number of DEGs (**B**), upregulated DEGs (**C**), and downregulated DEGs (**D**) at each time point.

To identify differentially expressed genes (DEGs) relative to the T0 time point, we calculated the fold change in fragments per kilobase of transcript per million (FPKM) values for each sample over that of T0. Genes fulfilling the criteria of |Log_2_(fold change FPKM)| > 1 and adjusted *p*-value < 0.05 were defined as DEGs. We obtained 10,048 DEGs out of the 18,883 mapped genes across all pairwise comparisons. Specifically, the numbers of DEGs identified at the T1, T3, T6, and T24 time points were 4477, 7728, 6191, and 2460, respectively (Figure 1A). We detected the highest number of DEGs at T3, followed by T6, T1, and T24. This pattern indicates that the influence of MeJA application and wounding reaches its maximum at 3 h after the onset of MeJA treatment and wounding. We observed the same pattern for upregulated and downregulated DEGs.

We further analyzed the DEGs of four time points in a Venn diagram (Figure 1B) that allowed us to find and exclude genes counted as DEGs at different time points. We detected the greatest number of DEGs at the area of T3 and T6, with 1838 genes, followed by DEGs detected at T3 alone (1748). Of the 10,048 DEGs, 1035 were differentially expressed across all four time points compared to T0. We plotted the corresponding Venn diagrams for upregulated and downregulated DEGs across the four time points (Figure 1C,D), which revealed a similar trend. There is also a case where it can be called ‘mixed pattern DEGs’. This indicates DEGs that were upregulated at one time point but downregulated at another time point. For example, if there is a gene that is upregulated at T1, but downregulated at T3, this gene is found in the T1-T3 co-zone (848, 8.4%) in Figure 1B. This gene is also counted as one of the genes belonging to T1-only (527, 11.0%) and T3-only (1098, 9.7%) regions in Figure 1C,D, respectively. There were 350 mixed-pattern DEGs discovered in this study, which appears to be because responses to stimulus occur through complex processes. The regulation of expression levels of these genes over a time period appears to be worthy of an independent research theme.

### 2.2. GO and KEGG Enrichment Analysis for Functional Classification of DEGs

**Figure 2:**
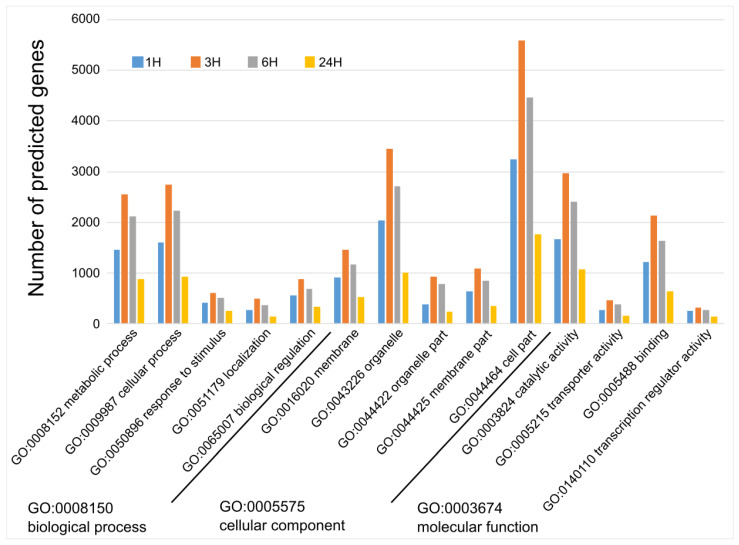
Gene ontology term enrichment analysis of differentially expressed genes. The three major categories of biological process, cellular component, and molecular function are shown at level 2.

**Figure 3:**
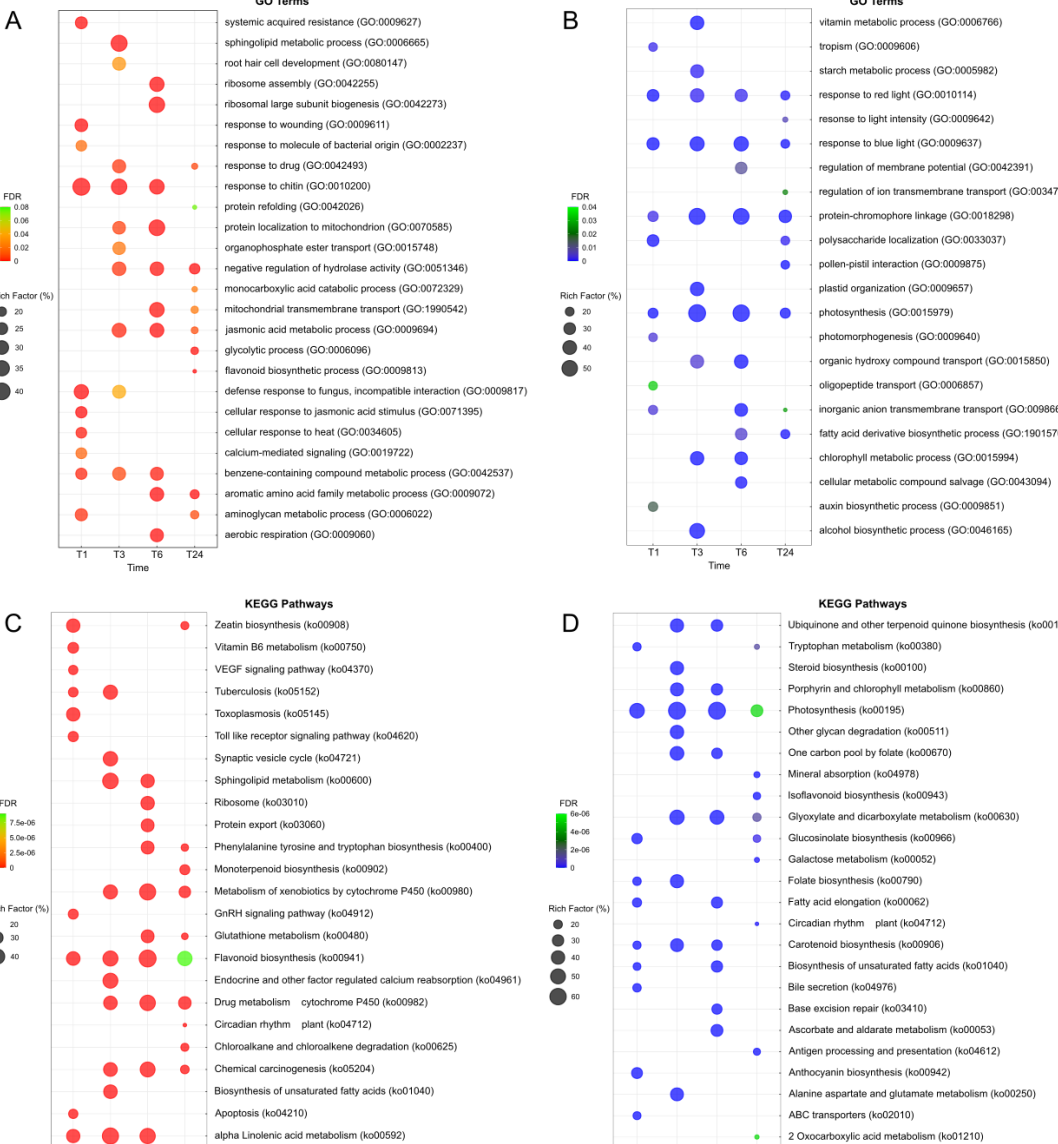
Enrichment analysis of differentially expressed genes in *S. tora* leaves after MeJA treatment and wounding. Gene ontology enrichment analysis of upregulated DEGs (**A**) and downregulated DEGs (**B**). Kyoto Encyclopedia of Genes and Genomes (KEGG) pathway enrichment analysis of upregulated DEGs (**C**) and downregulated DEGs (**D**). The results of enrichment analysis are shown for the top 10 gene rich factors at each time point. The rich factor is the ratio (%) of the number of DEGs assigned to each GO term (or KEGG pathway) relative to the number of genes assigned to that GO term (or KEGG pathway). The size of the dot indicates the gene rich factor. The color of the dot represents the false discovery rate (FDR) values. All *p*-values were below 0.05. The dot plots were generated using the ggplot function in the R package ggplot2.

We performed a gene ontology (GO) term enrichment analysis on the DEGs to predict their functions following MeJA treatment and wounding. We identified 14 GO terms (level-2) containing more than 5% of DEGs in each of the three key categories (level-1, biological process; 6718, cellular component; 8033, and molecular function; 7689 genes). As shown in Figure 2, the number of GO terms is proportional to the number of DEG. The number of genes associated with a significant GO term at the T3 time point was higher than at any other time point. Among the three key categories, we further analyzed the GO terms for the biological process category to predict gene functions in detail. To this end, we separately analyzed upregulated and downregulated DEGs (*p*-value cut off < 0.05), as shown in Figure 3A and Figure 3B, respectively. We selected the top 10 GO terms with the highest rich factor (%) for each time point.

We determined that the upregulated DEGs at T1 (Figure 3A) are related to defense responses, such as ‘systemic acquired resistance’ (GO:0009627), ‘response to wounding’ (GO:0009611), ‘response to molecule of bacterial origin’ (GO:0002237), ‘response to chitin’ (GO:0010200), ‘defense response to fungus, incompatible interaction’ (GO:0009817), ‘cellular response to jasmonic acid stimulus’ (GO:0071395), ‘cellular response to heat’ (GO:0034605), and ‘calcium-mediated signaling’ (GO:0019722). We speculate that this pattern reflects the rapid response of plants to externally applied MeJA or to wounding. At later time points (T3, T6, and T24), the GO term ‘jasmonic acid metabolic process’ (GO:0009694), which includes numerous JA biosynthetic genes, was highly enriched after external MeJA application. We also noticed the enrichment of DEGs at the T24 time points for the GO term ‘flavonoid biosynthetic process’ (GO:0009813). A similar GO analysis with downregulated DEGs (Figure 3B) revealed that GO terms related to photosynthesis are highly represented at all time points: ‘response to red light’ (GO:0010114), ‘response to blue light’ (GO:0009637), ‘photosynthesis’ (GO:0015979), ‘protein-chromophore linkage’ (GO:0018298), ‘chlorophyll metabolic process’ (GO:0015994), ‘photomorphogenesis’ (GO:0009640), and ‘response to light intensity’ (GO:0009642). A decrease in photosynthetic activity following exposure to biotic and abiotic stresses was reported [38,39]. 

We also performed a Kyoto Encyclopedia of Gene and Genomes (KEGG) pathway enrichment analysis to obtain additional information about the gene functions. This analysis indicated that upregulated DEGs are associated with the KEGG pathway ‘flavonoid biosynthesis’ (ko00941) at all time points (Figure 3C). Moreover, downregulated DEGs were related to the KEGG pathway ‘photosynthesis’ (ko00195), consistent with the GO term analysis above (Figure 3D). Thus, GO and KEGG enrichment analyses revealed that genes associated with flavonoid biosynthesis are significantly upregulated, while those related to photosynthesis are downregulated, in response to wounding and MeJA treatment.

### 2.3. Expression Analysis of the Flavonoid Biosynthetic Genes and Quantification of the Flavonoids Kaempferol and Quercetin

**Figure 4:**
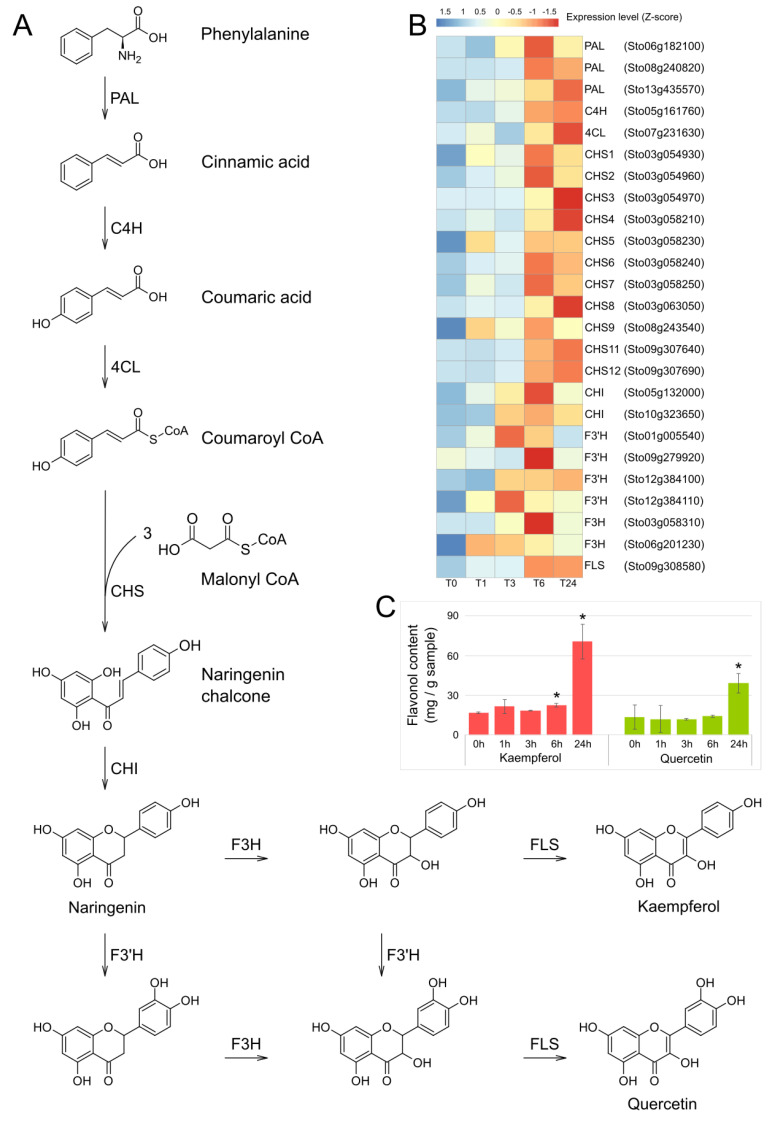
Expression analysis of genes involved in flavonoid biosynthesis induced after MeJA treatment and wounding in *S. tora* leaves. (**A**) Flavonoid biosynthesis pathway in plants. PAL, phenylalanine ammonia-lyase; C4H, cinnamate−4−hydroxylase; 4CL, 4−coumarate−CoA ligase; CHS, chalcone synthase; CHI, chalcone isomerase; F3′H, flavonoid 3′-monooxygenase; F3H, flavanone 3−hydroxylase; and FLS, flavanol synthase. (**B**) Heatmap representation of the expression of genes involved in flavonoid biosynthesis. The FPKM (fragments per kilobase of transcript per million mapped reads) values for each gene were Z-score normalized. The heatmap was plotted using the R package pheatmap. (**C**) Kaempferol and quercetin contents in *S. tora* leaves at the indicated time points. Asterisk indicates significant differences from T0, with *p*-value < 0.05 (Student’s *t*-test).

We investigated the transcript levels of genes encoding enzymes involved in flavonoid biosynthesis pathway (ko00941). We determined that 25 genes were the upregulated DEGs in the pathway. In the flavonoid biosynthesis pathway [40,41,42], illustrated in Figure 4A, 4-hydroxycinnamoyl CoA is generated through the phenylpropanoid biosynthetic pathway using phenylalanine as a substrate, and then three manlonyl CoAs are successively added to form a chalcone that forms the basic skeleton of flavonoids. This is the first committed step and is catalyzed by CHS. Among the 12 *CHS* genes of the *S. tora* genome [37], 11 *CHS*s, except *CHS10*, were upregulated DEGs after MeJA treatment and wounding. In particular, *CHS5*, *CHS6*, and *CHS7* showed significantly increased expression levels and reached maximum at T6. The flavonoid biosynthesis genes showed their maximum FPKM values mostly at T6 or T24 (Figure 4B and Supplementary Table S2). In addition, we quantified the levels of kaempferol and quercetin in the same samples; we observed a marked increase at T24 (Figure 4C). Together, these results suggest that kaempferol and quercetin are synthesized in response to the abundant expression of genes encoding flavonoid biosynthesis enzymes at T6 and/or T24, reaching a maximum accumulation at T24. Our results from this experiment are consistent with those of many previously published studies reporting that wounding [43,44] or MeJA treatment [45,46,47,48] increased the content of flavonoids.

### 2.4. Expression Analysis of the Jasmonic Acid Biosynthetic Genes

**Figure 5:**
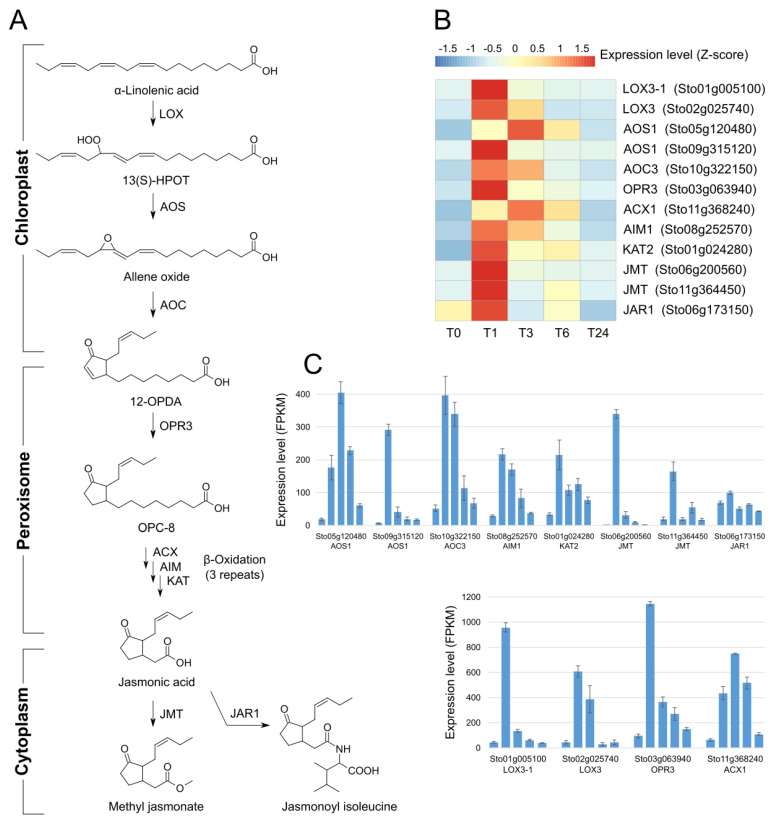
Expression analysis of genes involved in jasmonate biosynthesis following MeJA treatment and wounding in *S. tora* leaves. (**A**) Jasmonate biosynthesis pathway in plants. LOX, lipoxygenase; AOS, allene oxide synthase; AOC, allene oxide cyclase; OPR3, OPDA reductase, ACX, acyl−coenzyme A oxidase; AIM, ABNORMAL INFLORESCENCE MERISTEM (fatty acid methyltransferase); KAT, 3−ketoacyl−CoA thiolase; JMT, jasmonic acid carboxyl methyltransferase; JAR1, jasmonate resistant 1. The FPKM values for each gene were Z−score normalized. (**B**) Heatmap representation of the expression of genes involved in jasmonate biosynthesis. (**C**) Expression levels of JA biosynthesis genes.

JA biosynthesis starts in the chloroplast and is completed in the peroxisome; it is then modified into MeJA or JA-Ile in the cytoplasm (Figure 5A) [8,49]. Most JA biosynthetic genes were upregulated at T1 and some remained upregulated at T3 (Figure 5B,C and Supplementary Table S3). Application of exogenous MeJA and wounding dramatically increased the expression of two JMT genes at T1. This observation indicates that externally added MeJA and/or wounding influences the biosynthesis of endogenous MeJA. The *JAR1* gene encoding the enzyme that conjugates isoleucine to JA [50] was also strongly expressed at T1. This finding may reflect the need for JA-Ile to propagate signals for internal transmission [51] as well as MeJA to be released outside the plant.

### 2.5. Expression Analysis of the Ethylene, Salicylic Acid, and Abscisic Acid Biosynthetic Genes

**Figure 6:**
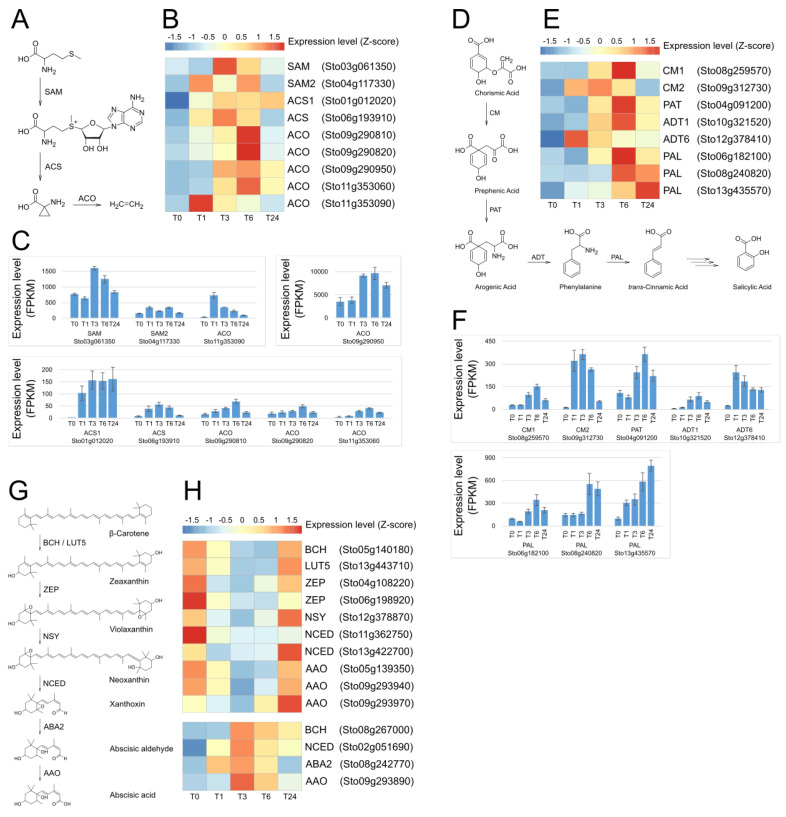
Expression analysis of genes involved in ethylene, salicylic acid (SA), and abscisic acid (ABA) biosynthesis following MeJA application and wounding. (**A**) Ethylene biosynthesis pathway. (**B**) Heatmap representation of the expression of genes involved in ethylene biosynthesis. (**C**) Expression level in FPKM of ethylene biosynthesis genes. (**D**) Salicylic acid biosynthesis pathway. (**E**) Heatmap representation of the expression of genes involved in salicylic acid biosynthesis. (**F**) Expression level in FPKM of salicylic acid biosynthesis genes. (**G**) Abscisic acid biosynthesis pathway. (**H**) Heatmap representation of the expression of genes involved in abscisic acid biosynthesis. SAM, S−adenosylmethionine synthase; ACS, 1−aminocyclopropane−1−carboxylate synthase; ACO, 1−aminocyclopropane−1−carboxylate oxidase; CM, chorismate mutase; PAT, prephenate aminotransferase; ADT, arogenate dehydratase; PAL, phenylalanine ammonia-lyase, BCH, beta-carotene hydroxylase; ZEP, zeaxanthin epoxidase; NSY, neoxanthin synthase; NCED, 9−cis−epoxycarotenoid dioxygenase; ABA2, ABA DEFICIENT 2 (xanthoxin dehydrogenase); and AAO, abscisic aldehyde oxidase.

Ethylene [52,53,54], salicylic acid (SA) [55], and abscisic acid (ABA) [56,57] are also important phytohormones that regulate growth, development, and response to biotic and abiotic stresses. Hence, we investigated the expression tendencies of the biosynthesis genes of these hormones over time periods. Unlike JA biosynthesis genes that showed immediate increases in expression level and maximum at T1, the genes of ethylene (Figure 6A–C and Supplementary Table S3) and salicylic acid (Figure 6D–F and Supplementary Table S3) biosynthesis pathways showed maximum expression levels at T6. Ethylene, SA, and jasmonates, including MeJA, form the basis of plant defense systems [58]. It was reported that MeJA induces the ethylene biosynthesis organ specifically [59], and that SA biosynthesis has an inverse correlation with MeJA [60]. RNA-seq analysis in this study also suggests that the biosyntheses of ethylene and SA occur after endogenous MeJA biosynthesis induced by exogenous MeJA treatment and/or wounding. Although research on cross-talk between them is in progress, much effort is still needed to fully understand it.

Unlike ethylene and SA, the expression pattern for ABA biosynthetic genes was complicated and showed two opposite expression patterns after wounding and MeJA treatment. We identified 14 ABA biosynthesis DEGs of which 10 DEGs were downregulated at T1, and the remaining four were upregulated (maximum at T3), as shown in Figure 6H and Supplementary Table S3. It is clear that wounding or MeJA treatment affected the expression of these genes, as the expression levels at T1 or T3 were significantly increased or decreased compared to T0. However, we were unable to elucidate their correlation in this study. Previous references reported that ABA biosynthesis was not the primary response to wounding [61,62]. In addition, it is also known that endogenous MeJA secretion [63] or external MeJA treatment [64] induces ABA biosynthesis under water-deficient conditions. 

### 2.6. Anthraquinone Biosynthesis and Expression of CHS-Ls

*S. tora* seed contains a useful medicinal compound, anthraquinone, which has anti-inflammatory, anti-tumor, and anti-fungal properties [17,25,65]. In our previous study [37], we found 16 *CHS-L* genes and showed that CHS-L9 is the enzyme responsible for the biosynthesis of anthraquinone in *S. tora* seeds. In the underlying biochemical reaction, CHS-L9 converts eight malonyl CoA molecules to produce atrochrysone carboxylic acid, a precursor to various anthraquinones.

**Figure 7:**
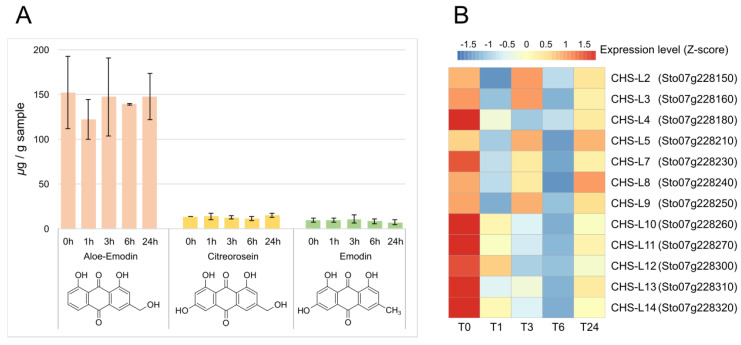
Content analysis of anthraquinone and expression pattern of *CHS*−*Ls*. (**A**) Anthraquinone contents in *S. tora* leaves after MeJA treatment and wounding. (**B**) Heatmap representation of the expression of *CHS*−*L* genes. FPKM values were Z−score normalized and visualized as a heatmap with pheatmap.

In this study, we investigated anthraquinone contents in *S. tora* leaves. We detected experimentally significant levels of aloe-emodin, emodin, and citreorosein, and their amounts varied little over the entire time points including T0. Moreover, emodin and citreorosien were less than 1/10 of aloe-emodin (Figure 7A). Anthraquinones are mainly found in the seeds, so it is not surprising that small amounts of limited types were found in the leaves. However, sennosides, which are dimers of dianthrone glucosides, are mainly found in leaves [66]. Sennoside A and B are homodimers of rhein-9-anthrone glucosides, while sennosides C and D are heterodimers of a rhein-9-anthrone glucoside and an aloe-emodin glucoside. Thus, rhein and aloe-emodin are the anthraquinones that can be found markedly in the leaves. However, we detected only aloe-emodin. The reason is that rhein has a carboxylic group substituent, thus basic aqueous methanol should were used to extract [67,68]. However, we used only methanol as an extraction solvent for the purpose of detecting other anthraquinones, which are neutral molecules, such as emodin, obtusin, obtusifolin, aurantio-obtusin, chrysophanol, and physcion, and these anthraquinones were also not detected, probably because of their low abundance in the leaves compared to seeds or roots.

RNA-seq analysis of 16 *CHS-L* genes, candidates for a central role in anthraquinone biosynthesis, explained the low abundance of these compounds in the leaves. Four were not expressed at all, and 12 *CHS-L* genes were downregulated (Figure 7B). 

We conclude that wounding and/or MeJA application acts adversely on the expression of anthraquinone biosynthesis genes in the leaves of *S. tora* and inhibits anthraquinone biosynthesis. The three anthraquinones detected in this experiment are considered to have been produced before the wounding and/or MeJA application and maintained during the subsequent time points. 

### 2.7. Validation of RNA-seq by qPCR Analysis of CHS and CHS-L Expression

We observed an increase in the content of flavonoids (Figure 4C) after MeJA treatment and wounding, whereas the levels of anthraquinones were not affected (Figure 7A). This result is in line with the RNA-seq data that show increased expression for *CHS*s, encoding a key enzyme in flavonoid biosynthesis, and decreased expression of *CHS-L*s, a likely candidate gene for anthraquinone biosynthesis. To verify the expression levels obtained by RNA-seq, we conducted qPCR analysis of selected genes. Specifically, we chose *CHS5*, *CHS6*, and *CHS7* for their highest FPKM levels. In addition, we also chose *CHS-L7*, *CHS-L8*, and *CHS-L9*, because *CHS-L9* was demonstrated to contribute to anthraquinone biosynthesis [37], and CHS-L7 and CHS-L8 are the two closest to CHS-L9 in their amino acid sequences [37]. 

**Figure 8:**
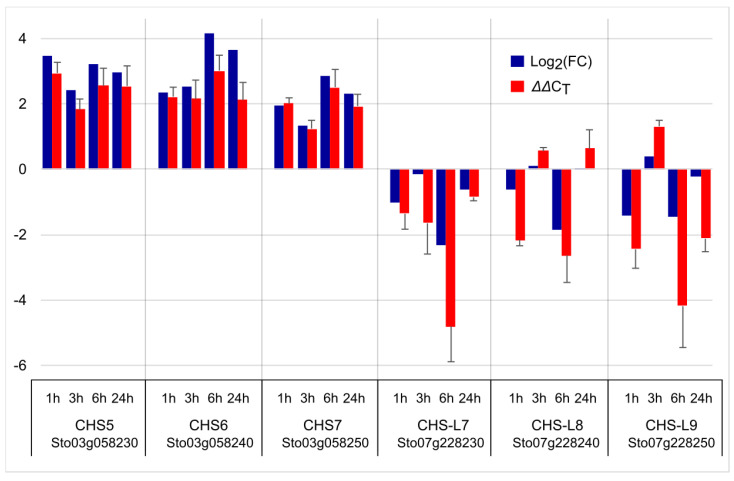
Confirmation of RNA−seq data by qPCR analysis. Log_2_(FC) (navy blue) represents RNA−seq data. FC is fold−change of FPKM values normalized to the T0 time point. ΔΔC_T_ (red) represents the increase or decrease from ΔC_T_ at T0.

The qPCR results (ΔΔC_T_) largely follow the same trend as the RNA-seq data, as upregulated genes in the RNA-seq data had positive Log_2_(FC) values that are accompanied by positive ΔΔC_T_ values; conversely, downregulated genes had negative Log_2_(FC) values and negative ΔΔC_T_, as summarized in Figure 8. The correlation between ΔΔC_T_ and Log_2_(FC) values was high and positive, with a coefficient of determination of R^2^ = 0.87 (Supplementary Figure S1 and Table S4). Therefore, these results show that our RNA-seq data are reliable.

## 3. Conclusions

In this study, RNA-seq analysis revealed a significant upregulation of flavonoid biosynthesis genes in response to both MeJA treatment and mechanical wounding. Biochemical analysis confirmed the high accumulation of kaempferol and quercetin 24 h post-treatment. These findings highlight the role of MeJA and wounding in enhancing flavonoid production. Conversely, genes related to anthraquinone biosynthesis, such as *CHS-L*, were downregulated, and levels of aloe-emodin, citreorosein, and emodin remained unchanged. We also observed the activation of genes involved in the biosynthesis of JA, MeJA, JA-Ile, ethylene, and SA. This study provides a method for linking transcriptomic data with secondary metabolite biosynthesis and lays the groundwork for future research to identify transcription factors regulating these pathways. The insights gained deepen our understanding of flavonoid and anthraquinone biosynthesis in *S. tora* under MeJA treatment and wounding.

## 4. Materials and Methods

### 4.1. Plant Materials and MeJA Treatment

*Senna tora* cv. ‘Myeongyun’ seeds were sown in a 32-well tray and grown for 2 weeks. Seedlings with similar cotyledon size were transferred to pots (300 mm diameter, 270 mm tall) and grown for an additional 8 weeks in a greenhouse (temperature 29 °C, humidity 38% under natural photoperiod from May to June at 35.8° N, 127.1° W, Jeounju, Republic of Korea). Control leaves (T0) were collected by cutting the petiolule of a compound leaf with scissors, then stored immediately in liquid nitrogen. Right after cutting the T0 leaves, 250 mL of MeJA (2 g/L) was sprayed onto whole plants. The MeJA solution was made by diluting the reagent (Sigma-Aldrich, Saint Louis, MO, USA, product number 392707) with sterilized distilled water. We collected individual leaflets from the same compound leaves after 1, 3, 6, and 24 h. The compound leaf of a healthy *S. tora* plant has six leaflets attached to one rachis. Each leaflet on one rachis was used for T0, T1, T3, T6, and T24. Twenty leaflets per time point for one plant were collected. Three plants for biological replicates were used. The harvested leaves were immediately frozen in liquid nitrogen, finely ground using a mortar and pestle (under liquid nitrogen condition), and then stored at −80 °C for RNA extraction. The ground samples were lyophilized for content analysis.

### 4.2. RNA Preparation and Sequencing

Total RNA was extracted from *S. tora* leaves using an RNeasy Plant Mini Kit (Qiagen, Hilden, Germany). Total RNA concentration was determined using a Quant-IT RiboGreen RNA Assay Kit (Thermo Fisher Scientific, Waltham, MA, USA) and its integrity was evaluated using TapeStation RNA ScreenTape (Agilent Technologies, Santa Clara, CA, USA). The RNA-seq libraries were constructed using high-quality RNA with an RNA integrity number (RIN) greater than 7.0, following the manufacturer’s protocol with an Illumina TruSeq mRNA Sample Prep kit (Illumina, San Diego, CA, USA). Library quantification was carried out using a qPCR Quantification Protocol Guide (KAPA Library Quantification kits for Illumina Sequencing platforms) and quality testing was performed with TapeStation D1000 ScreenTape (Agilent Technologies, Santa Clara, CA, USA). Library sequencing was conducted by Macrogen (Seoul, Republic of Korea) using a HiSeq4000 platform (Illumina).

### 4.3. RNA-seq Data Processing

HISAT v. 2.1.0 was used for alignment of the reads, and StringTie v. 1.3.4d was used for abundance estimation. Low-quality reads and adapter sequences were filtered out from the raw reads prior to processing, and then, the cleaned RNA-seq reads were mapped to the reference *S. tora* genome [37]. FPKM values for each annotated gene were calculated based on the number of mapped RNA-seq reads.

### 4.4. Statistical Analysis of the DEGs

The ratios of the FPKM values for the T1, T3, T6, and T24 time points to that of the T0 time point were defined as a fold change (FC). The genes with an FPKM value of zero at T0 were discarded. We selected genes of which |Log_2_(FC)| > 1 and an adjusted *p*-value < 0.05 as the DEGs. The statistical significance of the DEGs was determined using DESeq2 as described in our previously published work [36]. For each DEG set, the expression level in FPKM was converted to Z-scores by the pheatmap package.

### 4.5. GO and KEGG Enrichment Analysis

We carried out GO and KEGG enrichment analysis as shown in our previously published work [36]. For GO and KEGG enrichment analysis, we used the functional annotation information of genes in the reference *S. tora* genome provided in our previous study [37].

The DEGs were sorted into upregulated and downregulated DEGs ahead of enrichment analysis and then used separately. The rich factor for a specific GO term (or KEGG pathway) is determined by dividing the number of DEGs associated with that term (or pathway) by the total number of genes annotated for that term (or pathway). The results of upregulated and downregulated DEG groups are visualized in separate dot plots using the R package ggplot2. In both analyses, terms or pathways with *p*-values of 0.01 or less were selected and shown in Figure 3.

### 4.6. Flavonoid Content Analysis

Kaempferol and quercetin were extracted by vortexing the lyophilized leaf powder for 3 h in a methanol–chloroform mixture (1:1, *v*/*v*). The extract was passed through a 0.22-μm Whatman syringe filter (Whatman GmbH, Dassel, Germany) and subsequently concentrated under reduced pressure. Then, the concentrated samples were diluted 20 times with methanol prior to injection into high-performance liquid chromatography (HPLC). The HPLC analysis and data processing were performed using an Agilent 1100 HPLC system equipped with a Shimpack GIS-ODS column (4.6 × 250 mm, 5 μm) and Agilent ChemStation software (Agilent Technologies, Santa Clara, CA, USA). The oven temperature was set to 25℃. The gradient of eluent was made by a combination of solution A (HPLC-grade water and 0.0758% [*v*/*v*] trifluoroacetic acid) and solution B (HPLC-grade acetonitrile). The percentage of solution B over solution A was increased gradually from 10% to 50% for the first 8 min (from 0 to 8 min), increased to 70% at 14 min and 90% at 15 min, then maintained constant until 20 min. The flow rate was maintained at 1.0 mL/min. The compounds were confirmed by UV detection at 254 nm.

### 4.7. Anthraquinone Content Analysis

After sonicating the lyophilized leaf powder in 100% methanol, the mixture was centrifuged at 14,000 rcf for 15 min at 4 °C. The supernatant was collected and concentrated using a nitrogen gas evaporator. The residue was dissolved in methanol (1 mL), additional methanol was added to make 20 mL, and filtered with a 0.2 μm syringe filter. For quantitative analysis, samples were analyzed by electrospray ionization (ESI) using an UltiMate U3000-LTQ XL linear ion trap mass spectrometer system (Thermo Fisher Scientific). A 2 μL aliquot of the sample was applied to an ACQUITY UPLC HSS T2 C18 column (2.1 × 150 nm; 2.5 μm particle size; Waters, Milford, MA, USA), and the mobile phase used for the linear gradient consisted of 95–100% acetonitrile–water containing 0.1% (*v*/*v*) formic acid for 15 min and 100% acetonitrile for 5 min at a flow rate 0.3 mL/min. The quantitative data shown in this study were generated from triple technical replicates for each biological replicate. The mass spectrometry was analyzed using the Xcalibur^TM^ software (version 2.2 SP1.4; Thermo Fisher Scientific).

### 4.8. qPCR Analysis for CHS and CHS-L Expression

The same RNA samples used for RNA-seq were used for cDNA synthesis. Each RNA sample (1 μg) was employed for cDNA synthesis using amfiRivert cDNA Synthesis Platinum Master Mix (GenDEPOT, Baker, TX, USA). The cDNAs were diluted 10 times for qPCR templates and performed with a QuantStudio3 (Thermo Fisher Scientific). The reaction mix contained 1 μL diluted cDNA, 10 μL RbTaq^TM^ SYBR Green qPCR PreMix (Enzynomics, Daejon, Republic of Korea), 0.5 μM of each primer, and ddH_2_O in a final volume of 20 μL. The qPCR protocol was as follows: 95 °C for 10 min, 40 cycles of 95 °C for 15 s, 60 °C for 10 s, and 72 °C for 15 s. The specificity of the SYBR green PCR signal was confirmed with a melting curve analysis. All samples were analyzed in three biological replicates. Primers for *CHS5*, *CHS6*, *CHS7*, *CHS-L7*, *CHS-L8*, and *CHS-L9* were used. The elongation factor gene *EF1*-α of *S. tora* (Sto08g255550) was used as a reference gene. Primer information is given in Supplementary Table S5.

The comparative C_T_ method was applied to calculate ΔC_T_ and ΔΔC_T_ [69]. ΔC_T_ = C_T_ gene of interest − C_T_ reference gene, ΔΔC_T_ = ΔC_T_ gene at T1, T3, T6, or T24 − ΔC_T_ gene at T0. The reference gene was *EF1*-α. All C_T_, ΔC_T_, and ΔΔC_T_ values for each gene can be found in Supplementary Table S6.

## Data Availability

The RNA-Seq sequences generated from Illumina sequencing of *S. tora* were submitted to the National Center for Biotechnology Information (NCBI) Sequence Read Archive (SRA) database under the accession number SRR27718951-27718965.

## Supplementary Materials

The following supporting information can be downloaded at: https://www.mdpi.com/article/10.3390/plants13202944/s1, Figure S1: Linear correlation between Log2(FC) and *ΔΔ*C_T_; Table S1: Summary of RNA sequencing and mapping; Table S2: Expression analysis of genes involved in flavonoid biosynthesis induced by wounding and MeJA application; Table S3: Expression analysis of genes involved in plant hormones biosynthesis induced by wounding and MeJA application; Table S4: Correlation between Log2(FC) and ΔΔCT; Table S5: Primer information for qRT-PCR; Table S6: Summary of CT, ΔCT, and ΔΔCT values obtained from qPCR.
